# Supplementary material for: Effect of Rubus idaeus Extracts in Murine Chondrocytes and Explants
Source: Biomolecules. 2021 Feb 9;11(2):245. doi: 10.3390/biom11020245 (PMC7915036; doi:10.3390/biom11020245)
Supplement: Supplementary file 1 [file biomolecules-11-00245-s001.pdf]

Supplementary file

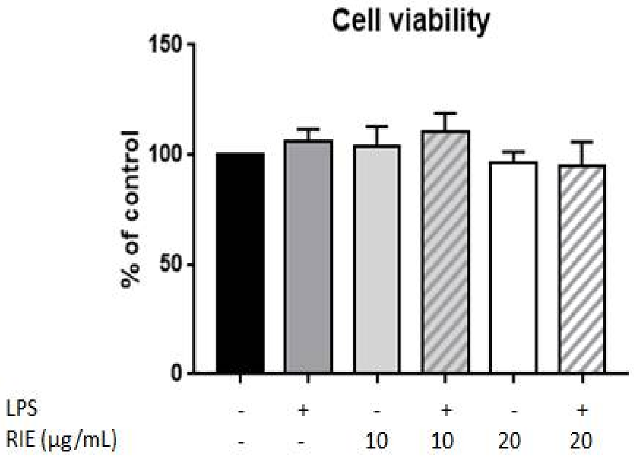

Supplemental Figure 1: Cell viability of macrophages does not change with treatment of LPS or RIE. The cell viability of macrophages is not altered with the pre-treatment of RIE (with 10 or 20 µg/mL) and with the stimulation of LPS (100 ng/mL). Data are mean±SEM from four experiments.
